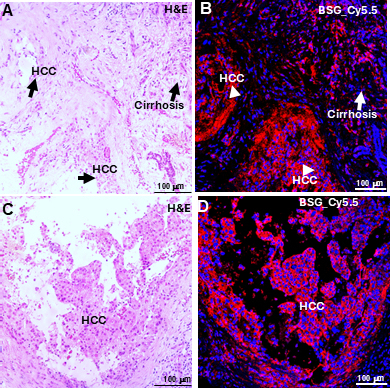# Multimodal Single-Cell Network Analysis Identifies Basigin/Cluster of Differentiation 147 as a Tumor-Associated Signaling Hub in Hepatocellular Carcinoma

**DOI:** 10.1016/j.gastha.2026.101050

**Published:** 2026-07-03

**Authors:** Thomas DW. Wang, Sangeeta Jaiswal, Eun-Young K. Choi, Shuo Feng, Hui Jiang, Thomas DS. Wang

**Affiliations:** 1 Department of Internal Medicine, University of Michigan, Ann Arbor, Michigan; 2 Department of Pathology, University of Michigan, Ann Arbor, Michigan; 3 Department of Biostatistics, University of Michigan, Ann Arbor, Michigan

**Keywords:** Hepatocellular Carcinoma, Single-Cell RNA Sequencing, hdWGCNA, BSG, CellChat

## Abstract

**Background and Aims:**

Hepatocellular carcinoma (HCC) ranks among the leading causes of cancer mortality globally, driven by late diagnosis and inadequate sensitivity of biomarkers like α-fetoprotein for early or indeterminate lesions. Single-cell RNA sequencing (scRNA-seq) enables precise mapping of tumor heterogeneity and early malignant transitions. This study aims to apply scRNA-seq analysis to detect clinically important molecular patterns that define the early stages of malignant transformation in HCC and facilitate the diagnosis of small or ambiguous lesions.

**Methods:**

Here, we integrated 2 independent scRNA-seq datasets (GSE149614 and GSE189903) from non-tumor and HCC tissues. Following batch correction, hepatocyte subpopulations were delineated via clustering, differential expression, pseudotime, and Cellular Trajectory Reconstruction Analysis using gene Counts and Expression analyses to reconstruct normal-to-malignant trajectories. High-dimensional weighted gene coexpression network analysis identified stage-correlated modules, while CellChat and protein-protein interaction networks revealed intercellular signaling. Candidate expression was validated in paired human liver specimens via quantitative immunofluorescence.

**Results:**

We uncovered a hepatocyte continuum with escalating stemness and oncogenic activation (MYC, E2F, G2M pathways). The Hep-M20 module, most strongly stage-associated, pinpointed Basigin (BSG)/cluster of differentiation 147 (CD147) as a hub gene with monotonic pseudotime upregulation and stemness correlation (ρ = 0.48, *P* < 2.2 × 10^−16^). CellChat exposed a cyclophilin (peptidylprolyl isomerase A [cyclophilin A]/peptidylprolyl isomerase B [cyclophilin B])-BSG/CD147 axis mediating tumor-fibroblast-T cell crosstalk. Ex vivo, BSG/CD147 showed elevated HCC expression (*P* = 2.9 × 10^−11^) with superior diagnostics (AUC = 0.93–0.96; sensitivity 86%–87%; specificity 93%–97%), even in <2 cm lesions.

**Conclusion:**

This multimodal analysis identifies BSG/CD147 as a tumor-associated signaling hub linked to hepatocyte state transitions and microenvironmental remodeling with potential utility for enhancing detection of small lesions in targeted imaging contexts.

## Introduction

Hepatocellular carcinoma (HCC) is a common primary liver malignancy and a leading cause of cancer-related deaths worldwide.^1^^,^^2^ The incidence continues to rise, driven by chronic hepatitis B and C infections, metabolic liver disease, and cirrhosis.^3^ Despite advances in imaging and therapy, most patients are diagnosed at an advanced stage when curative interventions, such as resection or transplantation, are no longer useful.^4^ Early-stage HCC is frequently asymptomatic, and current biomarkers lack the sensitivity and specificity needed for reliable detection.^5^ This limitation underscores the need for biologically based biomarkers that can identify early or indeterminate (Liver Imaging Reporting and Data System [LI-RADS] 3/4) lesions on imaging to improve clinical decision-making.^6^

Many imaging techniques, such as ultrasound, computed tomography, and magnetic resonance imaging, are used in clinical practice^7^ and serum biomarkers, such as α-fetoprotein and des-γ-carboxy prothrombin, are commonly used to assist in detection and monitoring. However, diagnosing HCC at an early stage remains a significant challenge. These techniques often lack sensitivity, especially with small lesions or in the context of liver cirrhosis.^8^^,^^9^ Furthermore, false-positive diagnoses may lead to unnecessary invasive procedures or undue patient anxiety. Therefore, molecular signatures, such as serum or tumor biomarkers that deliver higher sensitivity and specificity for the early and accurate detection of HCC as diagnostic tools, are greatly needed.

Tumor heterogeneity is a well-known feature of HCC, and results in high diversity at the cellular, molecular, functional, and lineage levels.^10^ Cellular and molecular variability poses a significant barrier to early diagnosis.^11^ This biological complexity gives rise to distinct molecular profiles even at early disease stages and in small nodules. Thus, conventional imaging phenotypes or bulk biopsy assessments may fail to capture the full molecular lesion complexity. In these contexts, molecular signatures, such as gene-expression panels, pathway-activation markers, or cell type–specific transcripts, may provide the sensitivity needed to detect small or early HCC lesions by capturing the molecular “footprint” of malignant transformation that precedes overt radiological changes.^12^

Traditional bulk transcriptomic methods suffer from aggregation of signals across all cells within the sample, thereby overlooking both rare populations and interactions among distinct cell types.^13^ In contrast, single-cell RNA sequencing (scRNA-seq) has revolutionized this landscape by enabling high-resolution transcriptomic analysis at the level of individual cells. This approach is capable of revealing rare hepatocyte and progenitor-like cells, reconstructing lineage trajectories, and delineating signaling networks that underlie cancer transformation. Although scRNA-seq has been used to profile immune and stromal HCC components, the full potential to identify clinically actionable progression-associated biomarkers remains unrealized. High-resolution data generated by scRNA-seq allows for the discovery of candidate early biomarkers, such as specific transcripts expressed only in emerging malignant subpopulations or in the supportive microenvironmental niche, which remain undetected in bulk transcriptomic analyses. The aim of the study was to apply scRNA-seq analysis to identify clinically relevant molecular signatures that capture the process of early malignant HCC transition and support the diagnosis of small or indeterminate lesions.

## Materials and Methods

### Data Acquisition From Single-Cell RNA Sequencing Datasets

Single-cell transcriptomic datasets were obtained from the Gene Expression Omnibus repository (https://www.ncbi.nlm.nih.gov/geo) to investigate gene expression dynamics during HCC transformation. Two independent datasets were evaluated. The discovery dataset (GSE149614) consisted of liver specimens from n = 10 patients with HCC distributed in 4 tissue types, including nontumor liver, primary tumor, portal vein tumor thrombus, and metastatic lymph nodes.^14^ Only cells from nontumor liver and primary tumor tissues were retained to focus on early cancer transformation. The cohort was predominantly male and spanned an age range of 48 to 66 years, with underlying etiologies including hepatitis B virus (HBV), hepatitis C virus (HCV), and nonviral causes. Patients represented multiple tumor-node-metastasis stages (I–IV). Detailed demographic and clinical characteristics have been previously reported (see Supplementary Table 1 in Reference 14). A validation dataset (GSE189903) was used for independent module preservation analysis and included n = 4 patients with HCC.^15^ The cohort included patients with diverse tumor sizes, and single cells were isolated from multiple tumor regions as well as adjacent nontumor liver tissue. Detailed clinicopathologic characteristics of the validation cohort, including patient demographics and tumor features, have been previously reported in the original publication (see Supplementary Table 1 in Reference 15). This approach enabled assessment of the robustness and reproducibility of gene coexpression network modules derived from the primary dataset across independent patient cohorts and sequencing platforms.

### Single-Cell RNA Sequencing Data Processing and Quality Control

All analyses were conducted using R software v4.4.0 with the Seurat v5 package.^16^ Raw gene expression count matrices were imported and converted into Seurat objects. Quality control procedures were applied to exclude low-quality cells using standard filtering criteria, including the removal of cells with high mitochondrial gene content that indicate apoptotic cells, abnormally low or high gene counts that suggest empty droplets or doublets, and low total RNA expression. GSE149614 was preprocessed, and only batch correction was performed. For GSE189903, cells with nCount_RNA <800, nFeature_RNA <500, or mitochondrial gene percentage >25% were excluded. These quality control steps ensured that only high-quality, biologically meaningful single-cell profiles were retained. Batch correction was performed to correct technical variations across patients and sequencing runs using the Seurat integration pipeline. Shared biological features were aligned across diverse datasets to minimize the influence of technical artifacts and ensure integrity for downstream analyses, and included clustering, differential gene expression, and pseudotime trajectory modeling.

### Cell Clustering, Annotation, and Characterization

After initial quality filtering, the FindVariableFeatures function was used to identify highly variable genes with a focus on those that contribute most to cellular heterogeneity. The data were then normalized and centered using ScaleData, and dimensionality reduction was performed using RunPCA. The appropriate number of principal components for downstream analysis was determined with the ElbowPlot function. A shared nearest-neighbor graph was constructed using FindNeighbors. Unsupervised clustering was performed with FindClusters to identify transcriptionally distinct cell populations. For visualization in 2D space, RunTSNE and RunUMAP were applied. Cluster-specific gene expression was evaluated using FindAllMarkers for global differentially expressed gene (DEG) analysis across all clusters, and FindMarkers for pairwise comparisons between selected clusters. Cell-type annotation was performed manually using canonical liver cell-type marker genes reported in the literature and established human liver single-cell transcriptomic atlases. Hepatocytes were identified based on the expression of well-established markers, including albumin, apolipoprotein C-I, apolipoprotein C-II, cytochrome P450 family 3 subfamily A member 5, and alpha-1-microglobulin/bikunin precursor, together with additional supporting markers described in prior studies. Only clusters identified as hepatocytes were retained to focus on tumor-associated changes. Seurat visualization functions, including dot plots, violin plots, and bar plots, were used to display gene expression patterns, assess cellular composition, and compare distributions across normal and tumor samples.

### Analysis of Stemness Using Cellular Trajectory Reconstruction Analysis using gene Counts and Expression

CytoTRACE (Cellular Trajectory Reconstruction Analysis using gene Counts and Expression) was used to infer stemness and differentiation potential across normal, protumor (PT), and tumor clusters.^17^ The preprocessed Seurat object from dataset GSE149614 was used for the CytoTRACE analysis. The filtered and normalized scRNA-seq data were converted into a gene expression matrix by extracting raw counts using the as.matrix() function (object[["RNA"]]@counts). This matrix served as input to the CytoTRACE() function from the CytoTRACE R package (v0.3.3) using default parameters unless otherwise specified. CytoTRACE scores, which reflect transcriptional diversity and infer cellular plasticity, were incorporated into the Seurat object metadata. These scores were visualized on Uniform Manifold Approximation and Projection embeddings to assess differentiation gradients across clusters. Higher CytoTRACE scores corresponded to less differentiated, more stem-like states and were used to inform downstream trajectory analyses.

### Pathway Enrichment Analysis

Single-cell pathway enrichment analysis was performed using the escape R package (v2.2.3) on a Seurat v5 object containing hepatocytes. Enrichment scores were extracted from the “escape.ssGSEA” assay and converted into a matrix with cells in rows and pathways in columns. Average enrichment scores per pathway were computed across normal, PT, and tumor groups using dplyr. Averaged data were visualized using pheatmap by applying row-wise scaling and hierarchical clustering to highlight differential pathway activity patterns among groups.

### Gene Coexpression Network Analysis

High-dimensional weighted gene coexpression network analysis (hdWGCNA) was performed to identify transcriptional programs associated with HCC transition. Hepatocyte subsets were first extracted from the integrated Seurat object. Using the hdWGCNA R package, a gene expression correlation matrix was computed, and a soft-thresholding power of 10 was selected to generate a scale-free weighted adjacency matrix.^18^ This parameter was chosen based on the standard approach in WGCNA, where the power was selected to approximate scale-free topology, a common property of biological networks. Specifically, the scale-free topology fit index and mean connectivity across a range of powers were examined. Power = 10 was the lowest value at which networks began to exhibit scale-free behavior (R^2^ > 0.85). The soft-thresholding power controls how strongly coexpression similarities (correlation values) are weighted in the adjacency matrix. Higher powers emphasize stronger correlations and suppressed weaker ones. This improved the reliability of module detection, leading to more biologically coherent gene modules. A topological overlap matrix was then constructed to improve network robustness by emphasizing high-confidence gene-gene interactions. Gene modules were identified using average linkage hierarchical clustering and were followed by dynamic tree cutting to define module boundaries. Genes that did not cluster into any module were assigned to the gray module and indicated low or nonspecific connectivity. Module eigengenes, representative expression profiles of each module, were correlated with key clinical features, including tissue origin (normal vs tumor), tumor stage, and viral infection status (HBV/HCV) to assess potential biological relevance. Hub genes were defined as genes with the highest intramodular connectivity using the GetHubGenes function and were used to identify key regulators within each module.

### Preservation of Key Gene Modules

Module preservation analysis was conducted using GSE189903 to assess the reproducibility of coexpression modules identified in the primary dataset (GSE149614). The GetModulePreservation function from the hdWGCNA package was employed to compute Z-summary scores, which integrate multiple metrics of module preservation and quality, including intramodular connectivity and network density. The PlotModulePreservation function was used to visualize module preservation, and display module size against Z-summary scores. Modules with Z-summary values > 10 were considered highly preserved and indicated strong reproducibility across datasets.

### Identification of Stage-Associated Biomarkers

A multistep integrative analysis was performed using gene coexpression modules and DEG data to identify robust biomarkers associated with HCC progression. First, hepatocyte-specific gene coexpression modules were generated using hdWGCNA. Modules were prioritized based on a strong correlation with clinical variables, such as tumor stage and tissue origin. A set of DEGs was generated by comparing gene expressions between tumor-derived and normal hepatocytes to refine candidate genes with functional relevance. The DEGs were then intersected with the gene members of prioritized modules to identify overlapping genes that were both differentially expressed and co-expressed within tumor stage-associated modules. To identify genes associated with tumor stage, DEG analysis was performed using the Seurat package with tumor stage set as the active identity class (Idents). DEG analysis was performed using the FindAllMarkers() function to restrict the analysis to a predefined list of intersecting genes. This approach identified stage-specific marker genes by comparing expression profiles across tumor stages. Expression dynamics were visualized using violin plots to allow for identification of genes that demonstrated progressive upregulation with increasing tumor stage.

### The Cancer Genome Atlas Analysis of Stage-Associated Gene Expression

Transcriptomic and clinical data for HCC were obtained from The Cancer Genome Atlas (TCGA) using the TCGAbiolinks R package. Raw gene expression data (HTSeq counts, spliced transcripts alignment to a reference workflow) were downloaded and processed using the GDCquery, GDCdownload, and GDCprepare functions. Gene-level expression was quantified, and Basigin (BSG)/cluster of differentiation 147 (CD147) expression was extracted using the corresponding Ensembl gene identifier. Only primary tumor samples were retained to focus on tumor-specific expression. Samples lacking valid stage annotations or gene expression were excluded from further analysis. A Spearman’s rank correlation was performed to assess the relationship between BSG/CD147 expression and tumor progression. Additionally, boxplots, violin plots, and smoothed regression lines were generated to visualize stage-wise expression differences. Outliers were identified and excluded using the interquartile range method for sensitivity analyses. Due to the limited number of Stage IV samples, visualizations were focused on stages I–III to ensure balanced group comparisons.

### Trajectory Analysis of Biomarker Expression

Pseudotime trajectory analysis was performed using the Monocle 2 R package to model the dynamic progression of hepatocyte transformation during HCC development.^19^ Hepatocyte clusters previously identified using Seurat were used to construct the trajectory. These clusters were selected to capture the continuum from early to advanced tumorigenic states. Monocle 2 was applied to order cells along a pseudotime axis and infer a developmental trajectory from transcriptomic changes across individual hepatocytes. Gene expression was projected onto the trajectory to investigate the transcriptional activation patterns of candidate biomarkers. Pseudotime expression analysis was then performed to assess the onset and progression of gene activation along the transformation continuum from normal to malignancy.

### Cell-Cell Communication Analysis

To infer and analyze intercellular communication networks, CellChat R package v1.6.11 was used.^20^ The normalized expression matrix and metadata (cell-type annotations) were used to create a CellChat object via createCellChat() function. The object was then subset to include only signaling-relevant genes using subsetData() function. Analysis used the built-in CellChatDB.human ligand-receptor interaction database. In addition, BSG/CD147-peptidylprolyl isomerase A (cyclophilin A) (PPIA) and BSG/CD147-peptidylprolyl isomerase B (cyclophilin B) (PPIB) interactions were manually incorporated to investigate their role in cell-cell signaling. These interactions were curated based on known protein-protein interaction (PPI) evidence, and were added to the database, including the ligand-target pair: BSG/CD147 (target); PPIA, PPIB (ligands), and signaling pathway name: BSG_PPIA_PPIB. Interactions were appended to the CellChatDB$interaction and CellChatDB$complex slots prior to data subsetting. The modified database was then used to subset the expression data with subsetData() and proceed with communication probability inference.

Communication probabilities were computed using computeCommunProb(), followed by computeCommunProbPathway() and aggregateNet(), to infer and summarize pathway-specific signaling networks. Only interactions involving cell types with sufficient representation (minimum 10 cells) were retained. Visualizations such as netVisual_circle(), netVisual_bubble(), and netAnalysis_signalingRole_network() were employed to explore outgoing and incoming signaling patterns. The contribution of each ligand-receptor pair to the pathway was quantified using netAnalysis_contribution().

### Protein-Protein Interaction Analysis

The BSG/CD147 PPI network was obtained from the Search Tool for the Retrieval of Interacting Genes/Proteins database (https://string-db.org) using a confidence score cutoff of 0.7. The network was visualized and analyzed in Cytoscape (v3.10.3). To assess context-specific interaction dynamics, expression-weighted PPI scores were calculated for normal, protumor, and tumor groups based on the average expression levels of interacting proteins and visualized using heatmap.

### Ex Vivo Validation of Target Expression in Hepatocellular Carcinoma

Tissue procurement and use in this study were conducted in accordance with institutional guidelines and approved by the Institutional Review Board of the University of Michigan (Approval No: HUM00248521). Formalin-fixed, paraffin-embedded human liver sections were obtained from the archived tissue bank in the University of Michigan Department of Pathology. Sections (5 μm thick) were cut, mounted on Superfrost Plus glass slides (Fisher Scientific), and deparaffinized. Antigen retrieval was performed in sodium citrate buffer prior to staining. Slides were blocked with 5% goat serum for 1 h at room temperature, followed by overnight incubation at 4 °C with monoclonal anti-BSG/CD147 antibody (MA529060, Invitrogen) at a 1:500 dilution. After 3X washes with phosphate-buffered saline containing Tween-20 (PBST, 3 min each), sections were incubated with a Cy5.5-conjugated secondary antibody for 1 h at room temperature. Slides were then washed 3X with PBST and mounted with 1.5 mm coverslips using ProLong Gold Antifade Reagent with 4′,6-diamidino-2-phenylindole (8961; Cell Signaling Technologies). Fluorescence images were acquired using a confocal microscope with 20X objective under identical exposure settings for tumor and background liver. Mean fluorescence intensities were quantified by placing 3 × 20 × 20 μm^2^ boxes entirely within liver tissues using custom MATrix LABoratory software (MathWorks, Inc), while avoiding regions of saturated signal. Adjacent sections were processed for routine pathology hematoxylin and eosin staining and independently evaluated by an expert liver pathologist (E-YKC). On each slide, the pathologist delineated the tumor region and adjacent background liver, which were then used to quantify the fluorescence signal.

### Statistical Analysis

Unless otherwise specified, default statistical tests were used for all R functions. DEG analysis was performed using the Wilcoxon rank sum test to assess statistical significance. Image quantification data were analyzed using GraphPad Prism, v10.4.1. Paired t-tests were applied where appropriate to assess differences between matched groups. Receiver operating characteristic (ROC) curve analysis was used to evaluate sensitivity and specificity. Linear regression was used to assess the relationship between tumor size and target-to-background (T/B) ratio, calculated as the fluorescence intensity of the tumor (T) divided by that of the surrounding background (B) liver. Spearman analysis was performed to determine correlation coefficients.

## Results

### Data Acquisition from Single-Cell RNA Sequencing Datasets

Single-cell transcriptomic analysis was performed to identify candidate biomarkers for early HCC detection. The discovery dataset (GSE149614) consisted of liver specimens from n = 10 patients with HCC. Only cells from nontumor liver and primary tumor were retained to focus on early cancer transformation. A validation cohort (GSE189903) was used for module preservation analysis. This dataset included n = 4 patients with HCC with diverse tumor stages, sizes, and locations and was used to evaluate robustness and reproducibility of gene coexpression patterns derived from the primary dataset across independent patient cohorts and technical platforms.

### Single-Cell RNA Sequencing Data Processing and Quality Control

**Supplementary Figure 1:**
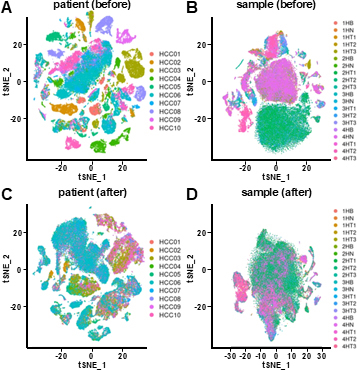


Initial analyses revealed substantial batch effects in both datasets with cells clustering primarily by patient origin and individual sample identifiers as shown on t-distributed stochastic neighbor embedding plots, Supplementary Figure 1A and B. Batch-driven clusters were eliminated using the Seurat integration pipeline. Postintegration, cells from different patients and samples exhibited extensive intermixing, and reflected successful alignment of biologically similar cell populations, Supplementary Figure 1C and D. This correction enabled reliable interpretation of cellular heterogeneity by ensuring that clustering and downstream analyses were driven by biological variations rather than technical differences. After stringent quality control and integration, the final datasets comprised 34,414 tumor-derived and 28,687 normal liver cells (GSE149614), and 43,656 tumor-derived and 30,614 normal liver cells (GSE189903).

### Cell Clustering, Annotation, and Characterization

#### Identification and annotation of liver cell types

**Supplementary Figure 2:**
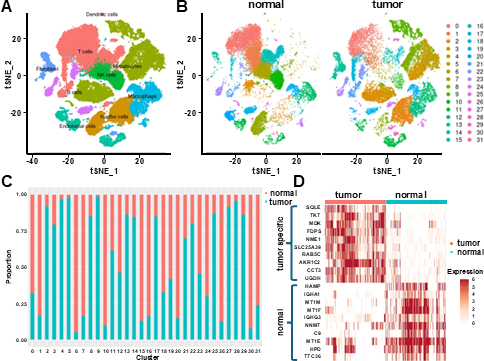


Unsupervised clustering identified 32 distinct cell clusters that represented a range of liver cell types (Supplementary Table 1). Annotations based on established marker genes revealed a diverse cellular composition, including hepatocytes, Kupffer cells, macrophages, dendritic cells, T cells, natural killer cells, B cells, fibroblasts, and endothelial cells, and reflects liver microenvironment heterogeneity, Supplementary Figure 2A. Stratification by tissue origin showed differential distribution of these populations between normal and tumor with several clusters enriched in either tissue type, Supplementary Figure 2B. Specific cell populations were preferentially represented in either normal (eg, cluster 6, 26) or tumor (eg, cluster 4, 5), Supplementary Figure 2C. DEG analysis revealed clear transcriptional differences between normal and tumor. Tumor hepatocytes showed upregulation of genes associated with malignancy, while normal hepatocytes retained expression of genes linked to physiological liver function, Supplementary Figure 2D. Clusters 3, 4, 5, 8, 9, and 26 were identified as hepatocytes based on marker gene expression.

#### Hepatocyte subpopulation analysis

**Figure 1:**
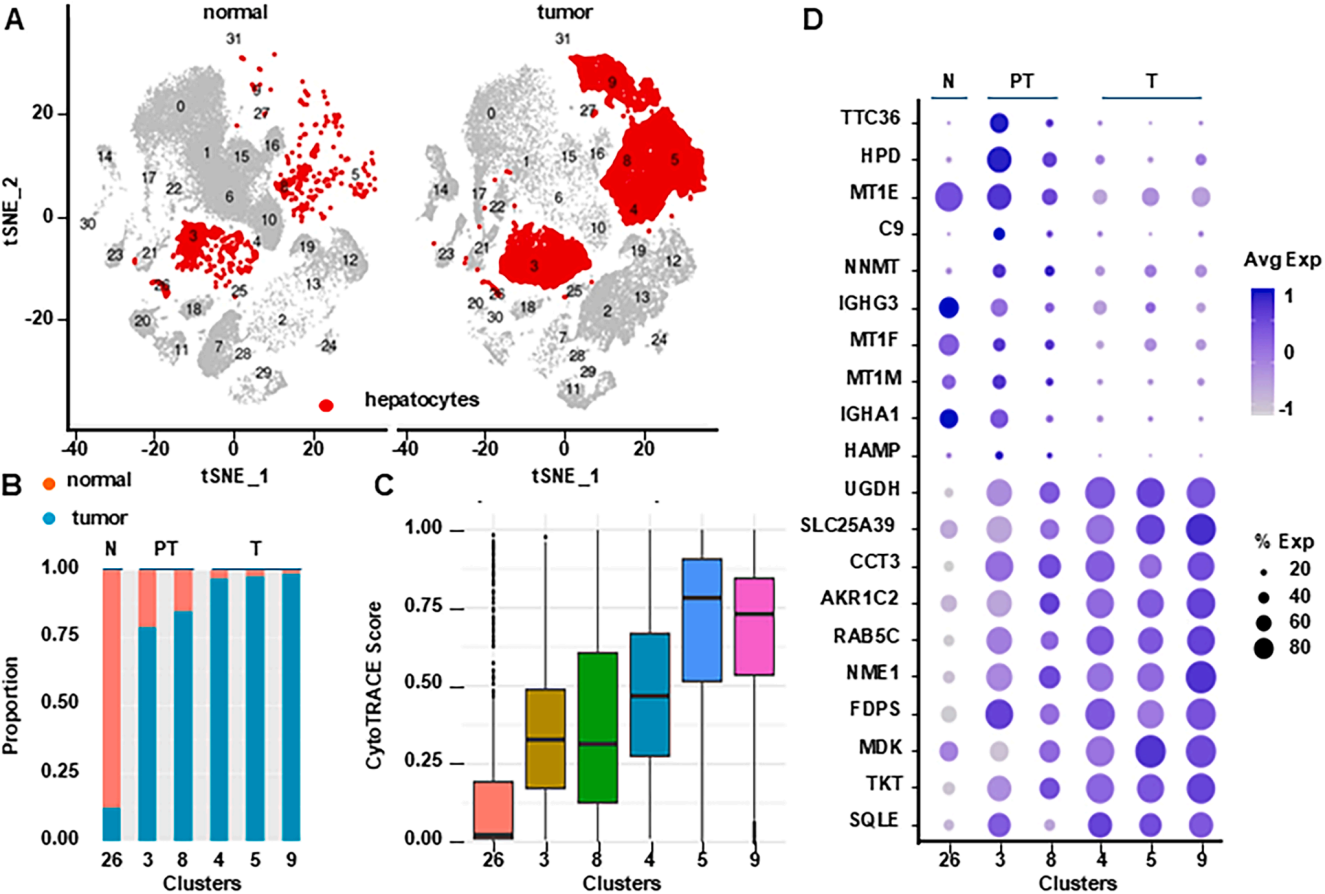
Hepatocyte subpopulations. (A) Single-cell clusters for normal and tumor specimens are shown. Hepatocyte clusters (red) were selected based on distinct gene expression patterns and demonstrated differential gene enrichment between normal and tumor. (B) Stacked bar plot illustrates proportional composition: 26 normal (N); 3 and 8 PT; 4, 5, and 9 tumor (T). Cluster 26 was composed predominantly of cells from normal liver (orange). Clusters 4, 5, and 9 showed marked enrichment in tumor (teal). Clusters 3 and 8 contained cells from both normal and tumor to reflect transitional states. (C) CytoTRACE analysis shows increase in stemness property from normal (cluster 26) to tumor (cluster 4, 5, and 9) cells. (D) Dot plot shows expression profiles for key differentially expressed genes across hepatocyte clusters. Dot intensity indicates average gene expression, while dot size represents percentage of cells expressing each gene in cluster. CytoTRACE, Cellular Trajectory Reconstruction Analysis using gene Counts and Expression; PT, protumor; tSNE, t-distributed stochastic neighbor embedding.

**Supplementary Figure 3:**
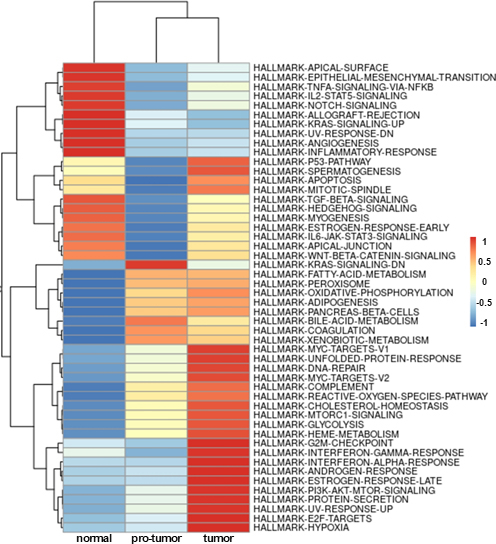


t-distributed stochastic neighbor embedding (t-SNE) analysis was used to explore hepatocyte heterogeneity between normal and tumor. Hepatocyte clusters (red) were identified based on distinct marker profiles, Figure 1A. Cluster composition analysis revealed differential enrichment levels. Cluster 26 was composed predominantly of normal hepatocytes (N), while clusters 4, 5, and 9 were enriched in tumor (T). Clusters 3 and 8 contained cells from both normal and tumor, designated as PT, and represented transitional cellular states, Figure 1B. CytoTRACE was used to evaluate stemness properties and was found to increase in tumor vs normal and PT clusters, Figure 1C. Dot plot analysis highlighted elevated expression of tumor-specific markers in clusters 4, 5, and 9, and expression of normal genes in cluster 26, Figure 1D. Clusters 3 and 8 exhibited a hybrid expression pattern by coexpressing both tumor and normal markers to support their role as intermediate states during tumor progression. Pathway enrichment analysis demonstrated significant upregulation of key cancer-related pathways within tumor clusters. Pathways such as MYC, which regulates cellular proliferation and metabolism,^21^ DNA repair, which maintains genomic integrity,^22^ the G2M checkpoint, which ensures proper cell cycle progression and prevents propagation of damaged DNA,^23^ and E2F targets, which drive DNA synthesis and cell cycle regulation,^23^ were all elevated, Supplementary Figure 3.

### High-Dimensional Weighted Gene Coexpression Network Analysis

**Figure 2:**
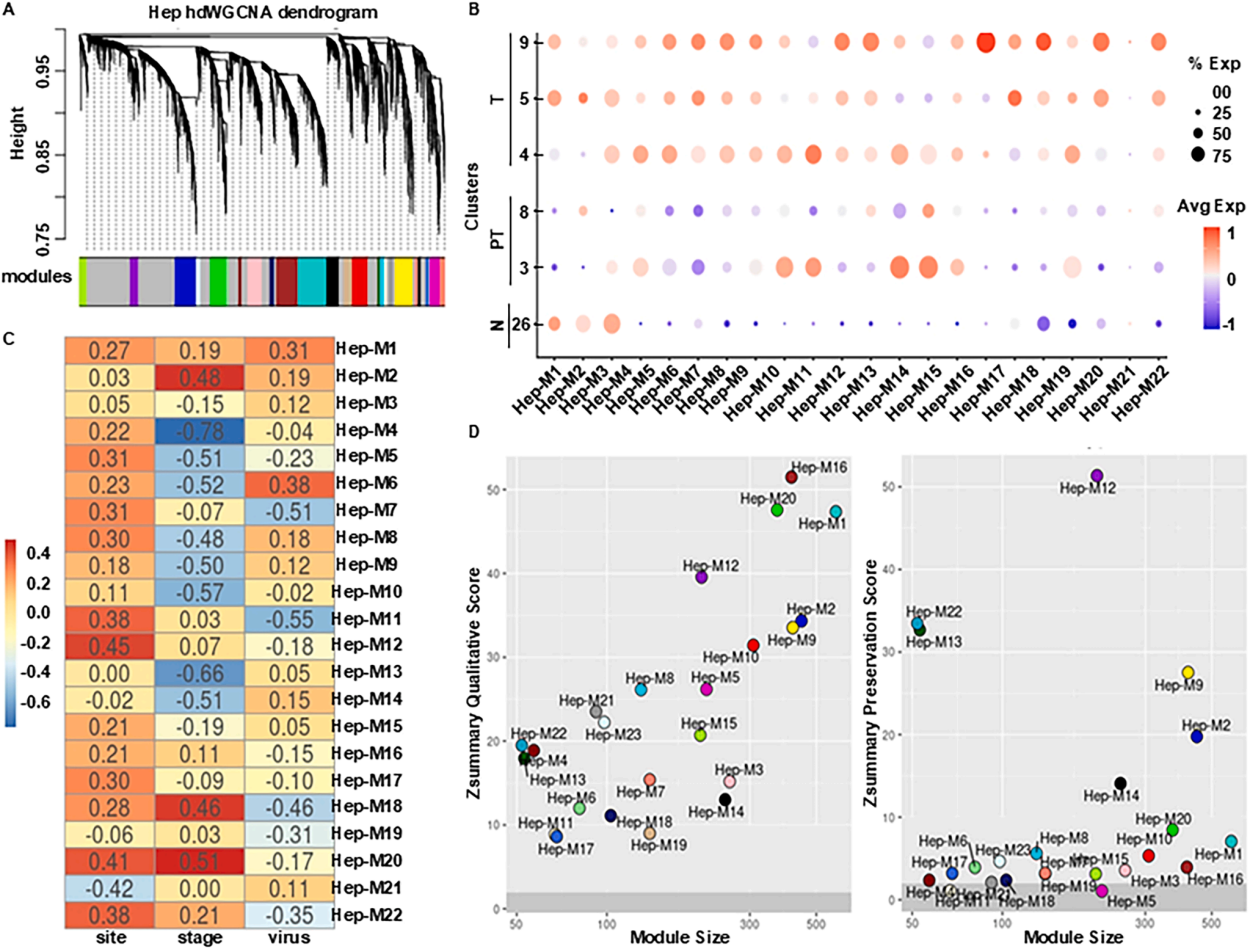
Gene modules associated with HCC progression. (A) hdWGCNA was used to generate a dendrogram that illustrates hierarchical gene clustering in distinct hepatocyte co-expression modules (depicted by unique colors). Genes not assigned are included in the gray module. (B) Dot plot shows gene expression for all modules identified (Hep-M1 to Hep-M22) across hepatocyte clusters. Dot size indicates proportion of cells within each cluster expressing genes from the respective module (% Exp) while color intensity reflects the average module expression level (Avg Exp). Modules, including Hep-M2, Hep-M18, and Hep-M20, exhibit progressively elevated expression from normal (26) through protumorigenic (3 and 8) and tumor-specific (4, 5, and 9). (C) Heatmap depicts correlations between module eigengenes (rows) and clinical features (columns), including tissue origin (“site”; normal vs tumor), pathological stage (“stage”), and viral infection status (“virus”). Positive correlations (red) indicate module enrichment with clinical features, and negative correlations (blue) represent inverse relationships. Module Hep-M20 demonstrates the strongest positive correlation (ρ = 0.51) with stage and highlights potential associations with tumor progression. (D) Module preservation analysis was performed using Z-summary statistics to demonstrate reproducibility and robustness of gene modules from the exploratory dataset (GSE149614) by comparison with the validation dataset (GSE189903). The Z-summary quality scores reflect internal consistency and connectivity, while the Z-summary preservation scores indicate module preservation. Modules with scores >10 (Hep-M2, Hep-M20) demonstrate strong preservation, and reinforce biological validity and significance across validation cohorts. hdWGCNA, high-dimensional weighted gene coexpression network analysis; PT, protumor.

**Supplementary Figure 4:**
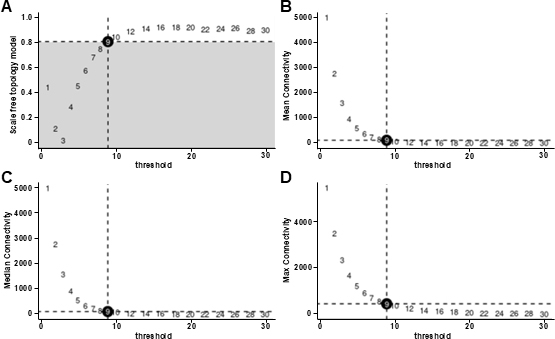


A soft-threshold value of 10 was chosen to build a gene network to best reflect natural biological relationships while keeping the connections simple, Supplementary Figure 4A-D. Using this network approach, 22 groups of genes that show similar activity patterns, called modules, and were identified among hepatocytes, Figure 2A. Genes that did not fit into any module were placed in a “gray” group and showed weak or inconsistent relationships with others. A dot plot shows how these gene modules change across hepatocyte clusters as they progress from normal to tumor cells, Figure 2B. Hep-M18 and Hep-M20 expression increased steadily from normal hepatocytes (cluster 26) to early tumor-like (clusters 3 and 8) and finally tumor-rich clusters (4, 5, 9). When comparing modules with clinical features, several meaningful patterns emerged. One group, Hep-M20, showed the strongest link to tumor stage (ρ = 0.51) to suggest a role in cancer progression. Hep-M12 was most associated with whether the tissue came from a normal or tumor, and Hep-M6 correlated most with viral infection status (HBV or HCV), Figure 2C. We note that modules with strong negative correlations may also represent biologically relevant processes; however, they were not prioritized in the context of marker discovery for progressive disease.

### Preservation of Key Gene Modules

Module preservation analysis was performed using hdWGCNA to assess robustness and reproducibility for coexpression modules across diverse conditions. GSE149614 was used as the discovery network, and GSE189903 was used to validate module preservation by applying the modulePreservation() function with 20 permutations. Zsummary.pres and Zsummary.qual statistics reflect preservation of density and connectivity, respectively, Figure 2D. Module 2 showed consistently high values in both Zsummary.pres and Zsummary.qual, indicating robust preservation of both density and connectivity across datasets. In contrast, Module 20 exhibited a strong Zsummary.qual signal but moderate Zsummary.pres values, suggesting that while gene-gene connectivity patterns are well conserved, overall network density is less strongly preserved. However, the Hep-M2 module did not exhibit a clear progression from normal to tumor (Figure 2B), and was not used to identify stage-associated biomarkers.

### Identification of Stage-Associated Biomarkers

**Figure 3:**
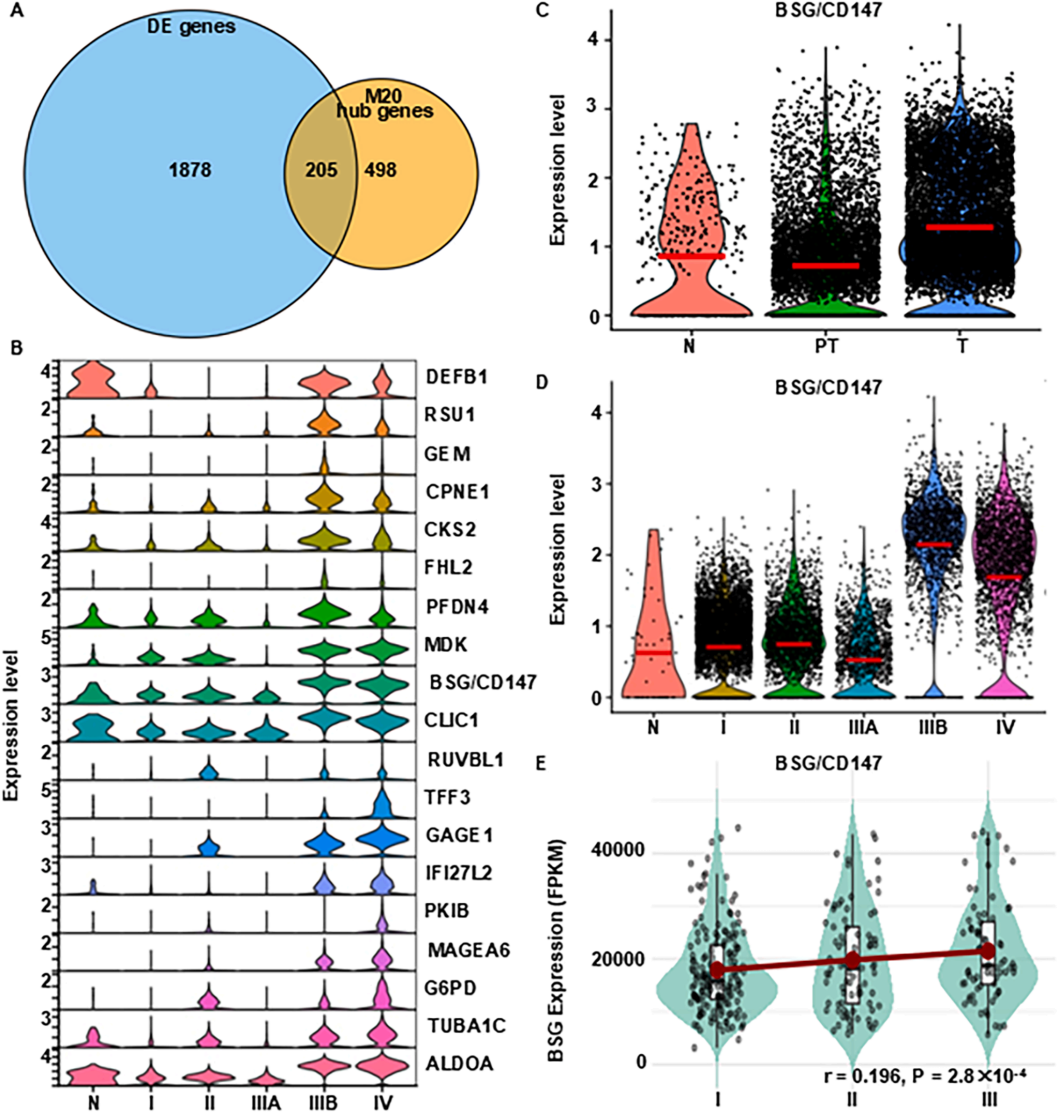
Identification of stage-specific HCC biomarkers. (A) Venn diagram illustrates intersection of differentially expressed genes between tumor and normal, and genes contained within module Hep-M20. This intersection identified 205 overlapping genes, and represents a group of candidate genes associated with tumor progression. (B) Violin plots show expression profiles of the top 18 overlapping genes from Hep-M20 across different pathological stages (N: normal; I, II, IIIA, IIIB, and IV). Distinct stage-dependent expression patterns emerge to highlight genes progressively upregulated with tumor progression. (C) Violin plots compare expression of BSG/CD147 in normal, PT, and tumor hepatocytes. (D) Stage-specific violin plots illustrate progressive increase of this biomarker across tumor stages from normal (N) through early-stage tumors (I-IIIA) to advanced stages (IIIB-IV) to underscore its potential as a progression-associated biomarker linked to tumor stage and hepatocyte transformation. (E) TCGA data analysis confirms positive correlation between BSG/CD147 and stage with Spearman’s coefficient ρ = 0.196, *P* = 2.8 × 10^−4^. BSG, Basigin; CD147, cluster of differentiation 147; FPKM, Fragments Per Kilobase of transcript per Million mapped reads; PT, protumor.

DEG analysis between normal and tumor hepatocytes were intersected with those from modules Hep-M20 to identify candidate biomarkers linked to HCC progression. This integrative analysis yielded 205 overlapping genes within Hep-M20, and represented a refined set of candidates likely involved in hepatocyte transformation, Figure 3A. Stage-specific DEG analysis was performed to identify the genes involved in tumor progression. Violin plots of the top 19 DEGs demonstrated stage-dependent upregulation with increased expression from normal liver to advanced tumor stages, Figure 3B. Among these, BSG/CD147 emerged as a promising candidate, and its location on the cell surface makes it an accessible and practical target for molecular imaging. Target expression was significantly elevated in tumor hepatocytes vs normal liver cells, Figure 3C. However, no significant differences were observed among early and intermediate stages (stage I–III), and stage III showed lower mean expression compared with normal liver. Overall, stage-stratified analysis did not reveal a consistent monotonic increase across tumor stages, Figure 3D. It should be noted that the relatively small number of normal samples may contribute to variability in baseline expression estimates and should be considered when interpreting these comparisons. TCGA data were analyzed to validate stage-associated BSG/CD147 expression. A progressive increase was observed with advanced pathology, Figure 3E. A significant positive correlation was found between BSG/CD147 expression and tumor stage in The Cancer Genome Atlas Liver Hepatocellular Carcinoma primary tumor samples.

### Trajectory Analysis of Biomarker Expression

**Figure 4:**
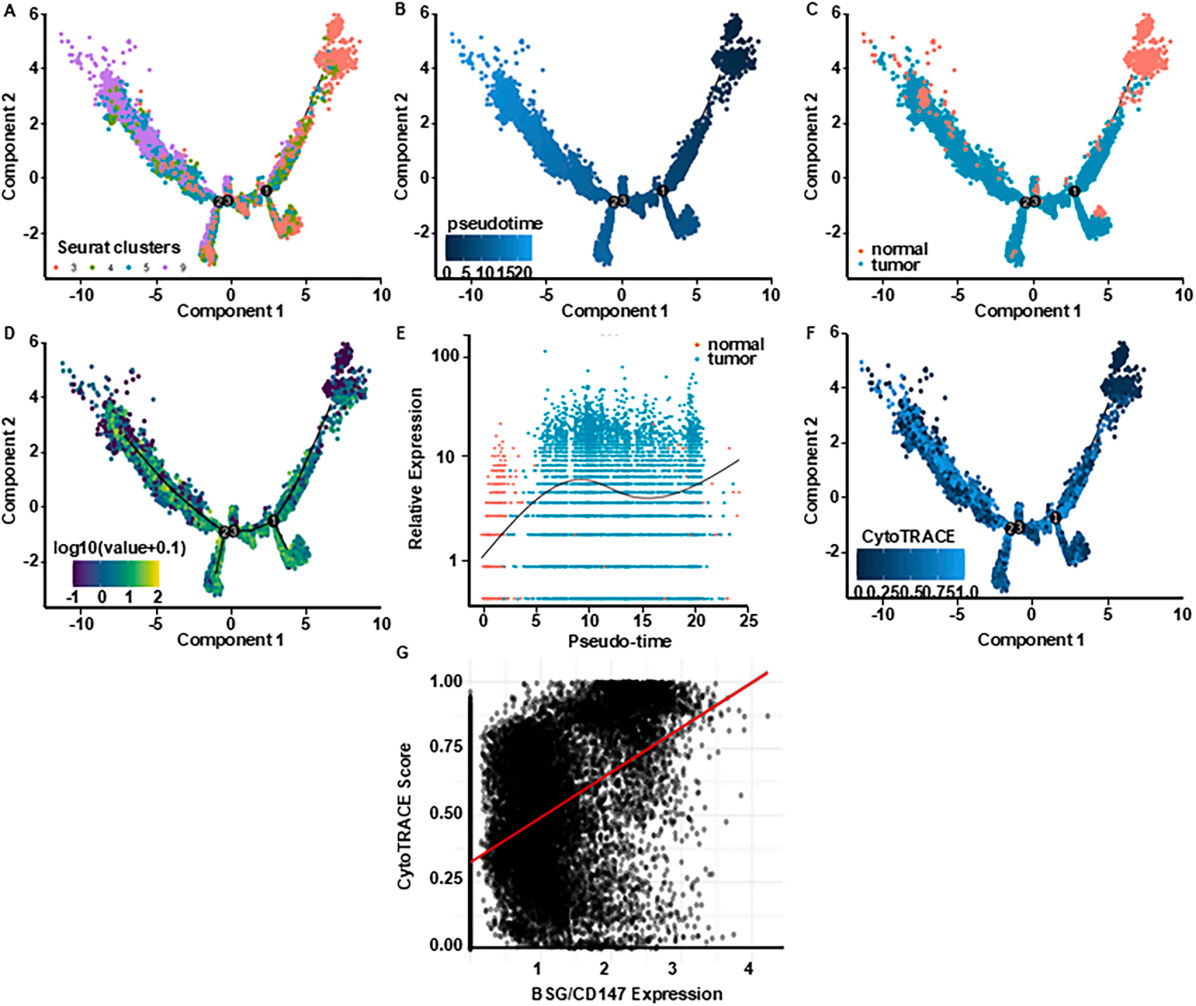
Pseudotime trajectory analysis. (A) Single-cell pseudotime trajectories display normal, PT, and tumor hepatocyte clusters (3, 4, 5, and 9) that capture a continuum of states from normal to early tumor and late stage tumor. (B) Pseudotime progression illustrates the inferred temporal hepatocyte ordering along the differentiation trajectory. Cells at the root (dark blue) represent early or transitional states, and cells at the terminal branches (light blue) correspond to more advanced tumor states. (C) Mapping of tissue origin (normal and tumor) onto the pseudotime trajectory highlights a clear transition from predominantly normal-derived hepatocytes at early pseudotime points to tumor-derived hepatocytes in later stages. (D) Expression profiles of BSG/CD147 are projected on the pseudotime trajectory. Color intensity indicates relative gene expression levels and reveals a marked increase in expression as cells progress toward advanced tumor stages. (E) Scatter plots demonstrate relative gene expression of BSG/CD147 over pseudotime to further support gene upregulation. Smoothed trend lines show a progressive increase in gene expression along pseudotime to support its role as a potential biomarker for HCC progression. (F) Projection of CytoTRACE score shows cells with low stemness potential situated at the trajectory beginning while cells with high CytoTRACE score were located at the trajectory end. (G) Scatterplot showing correlation between CytoTRACE score and BSG expression with Spearman’s coefficient r = 0.48, *P* < 2.2 × 10^−16^. BSG, Basigin; CD147, cluster of differentiation 147; CytoTRACE, Cellular Trajectory Reconstruction Analysis using gene Counts and Expression.

A single-cell pseudotime trajectory was constructed using Monocle 2 to model transcriptional dynamics during HCC progression. The trajectory began with cluster 3 (PT hepatocytes) at the root, and extended through clusters 4, 5, and 9 to represent progressively advanced tumor states, thereby forming a continuum from early to late tumorigenesis, Figure 4A. Cluster 8 was excluded from pseudotime analysis as it represents a transcriptionally distinct hepatocyte state enriched for acute-phase/stress genes (eg, *ORM1, ORM2, CLU*), which disrupted continuity of the hepatocyte-tumor trajectory defined by cluster 3. The arrangement of cells along pseudotime reflect their inferred developmental order with early-stage cells positioned near the root and advanced tumor cells occupying the terminal branches, Figure 4B. Mapping the tissue origin of cells onto the trajectory further supports biological relevance. Normal cells were located primarily at the early end of the pseudotime axis, while tumor-derived cells clustered at later stages to support a temporal transition from normal to malignant hepatocytes, Figure 4C. Gene trajectory plots demonstrated that BSG/CD147 expression increased progressively along the trajectory, Figure 4D. This trend was further supported by smoothed scatter plots that revealed sustained and gradual gene upregulation over pseudotime, Figure 4E. CytoTRACE scores projected onto the pseudotime trajectory revealed a gradual increase with lower scores observed at early stages and higher scores toward the terminal branches, Figure 4F. A significant positive correlation was observed between BSG/CD147 expression and stemness potential (Spearman’s coefficient ρ = 0.48, *P* < 2.2 × 10^−16^ to suggest that higher expression is associated with increased stemness, Figure 4G.

### Cell-Cell Communication Analysis

**Supplementary Figure 5:**
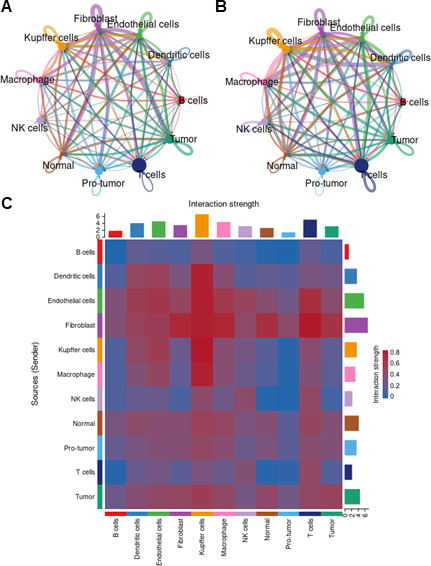


CellChat analysis was used to explore how different cell types communicate with each other. Key signaling pathways were identified using a database of known ligand-receptor pairs, and the strength of communication among cell groups was measured. Cell-cell communication patterns were visualized using CellChat, with interaction networks quantified by both interaction count, Supplementary Figure 5A and interaction weight, Supplementary Figure 5B. In the network map, thicker connecting lines represent more frequent or stronger interactions among cell types, Supplementary Figure 5A and B. Fibroblasts showed the highest level of communication activity, interacting with tumor cells (59 interactions, weight = 0.66), normal liver cells (53 interactions, weight = 0.70), and PT cells (40 interactions, weight = 0.41) to suggest a central role in shaping the tumor environment. A heatmap further illustrates the strength of signaling among different cell types, Supplementary Figure 5C. Overall, fibroblasts sent strong signals to immune cells, normal hepatocytes, and tumor cells to highlight their major role as communication “hubs” within the tissue.

**Figure 5:**
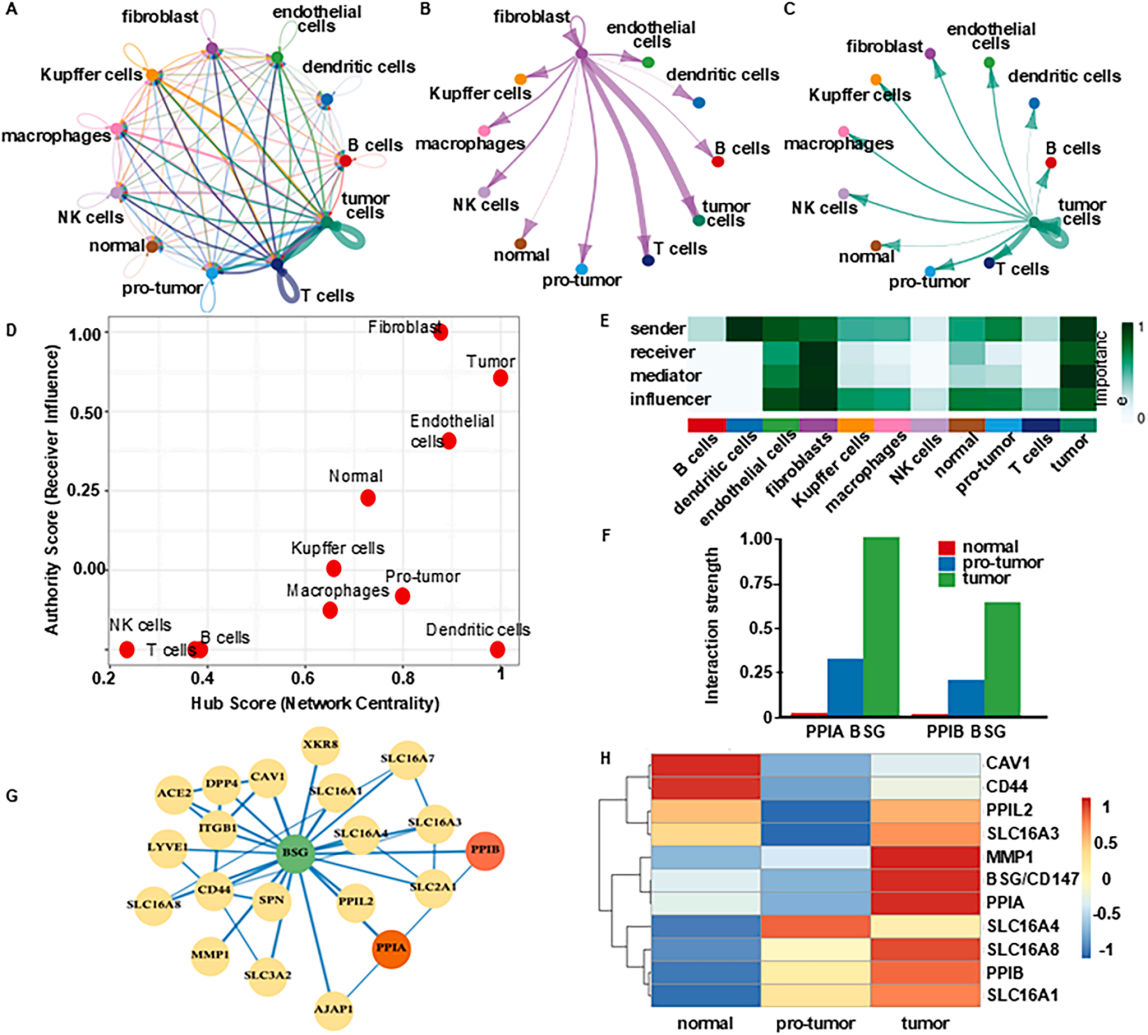
BSG/CD147 mediated cell-cell communication in tumor microenvironment. (A) NetVisual plot of BSG/CD147 signaling illustrates communication networks among cell types. Node size corresponds to cell group abundance. Edge thickness represents relative signaling probability. (B) Circle plot depicts the strength of fibroblast-derived signals to target cells that highlight significant communications with T and tumor cells. (C) Circle plot shows the strongest outgoing signaling from tumor cells toward T cells and indicates a dominant tumor-to-immune cell communication axis via BSG. (D) Authority vs hub scores reveal distinct cellular roles in BSG/CD147 signaling. Each point represents a cell population positioned according to its hub score (outgoing signaling strength) and authority score (incoming signaling strength). (E) Signaling role heatmap depicts cellular functions within the BSG/CD147 signaling network and displays the relative sending (hub) and receiving (authority) roles of each cell population. Tumor cells show strong hub activity to indicate a central role in initiating signaling, while fibroblasts exhibit dominant receiver activity. (F) Bar plot compares interaction strengths across normal, PT, and tumor conditions to demonstrate that tumor cells exhibit the highest signaling probability, followed by PT and normal cells. (G) PPI network was derived from STRING (confidence ≥0.7) and visualized in Cytoscape. Nodes represent proteins, and edges display predicted or validated interactions. BSG is highlighted in green as the central protein of interest, its canonical interacting partners PPIA and PPIB are shown in orange, and all remaining interacting proteins are shown in yellow to represent the extended interaction network. (H) Heatmap showing the expression-weighted PPI scores for BSG/CD147 across normal, PT, and tumor cells. Tumor cells exhibit the highest PPI activity, followed by PT and normal cells, to indicate enhanced BSG/CD147-mediated signaling during tumor progression. BSG, Basigin; PPIA, peptidylprolyl isomerase A (cyclophilin A); PPIB, peptidylprolyl isomerase B (cyclophilin B).

**Supplementary Figure 6:**
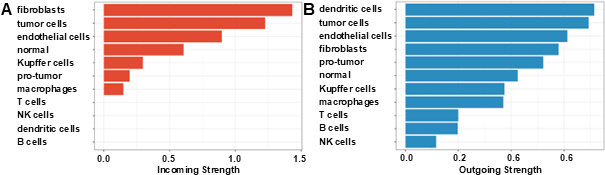


Analysis of cell-to-cell communication revealed that the BSG/CD147 signaling axis plays a major role in shaping the tumor microenvironment, Figure 5A. In this pathway, extracellular cyclophilins, primarily cyclophilin A (PPIA) and cyclophilin B (PPIB) act as secreted ligands, while CD147 (BSG), a transmembrane glycoprotein, functions as their receptor on target cells. Thus, high PPIA/PPIB expression defines mediator (sender) cells, whereas high CD147 (BSG) expression together with strong incoming signaling defines receiver/influencer cells. Among all cell types, dendritic cells sent out signals with greatest strength (0.71) and were identified as the main “sender” cells, while fibroblasts received the most signals (1.43) to become key “receivers” within the network, Supplementary Figure 6A and B. Strong communication was also observed from fibroblasts to both T cells and tumor cells, Figure 5B. The circle plot shows the strongest outgoing signaling from tumor cells toward T cells, highlighting a tumor-to-immune communication axis via BSG/CD147, Figure 5C. This inference is based on combined ligand and receptor expression and does not indicate that ligands or receptors are exclusively expressed by specific cell types. Accordingly, the inferred directionality does not imply strictly unidirectional signaling, as both cyclophilins (PPIA/PPIB) and CD147 (BSG) are expressed across multiple cell types. Tumor cells had the highest network “hub” score to highlight their central role, while fibroblasts ranked highest as signal receivers, Figure 5D. Overall, the analysis revealed distinct cellular roles as tumor cells function as communication hubs, fibroblasts as primary recipients, and immune and endothelial cells as important regulators within the BSG/CD147-cyclophilin signaling network, Figure 5E. The strength of cyclophilin A and B signaling (PPIA/PPIB) was further quantified across normal, pretumor, and tumor conditions, Figure 5F.

### Protein-Protein Interaction Analysis

BSG/CD147 was found to interact with several key proteins, including PPIA, PPIB, MMP1, and SLC16A1, to support its known role in matrix degradation and metabolic adaptation, Figure 5G. Network analysis identified PPIA and PPIB as hub interactors. Expression-weighted PPI scores revealed that tumor cells displayed the highest interaction strength, followed by PT and normal groups, to reflect a progressive activation of BSG/CD147-associated signaling during tumor development, Figure 5H.

### Ex Vivo Validation of Target Expression in Hepatocellular Carcinoma

**Figure 6:**
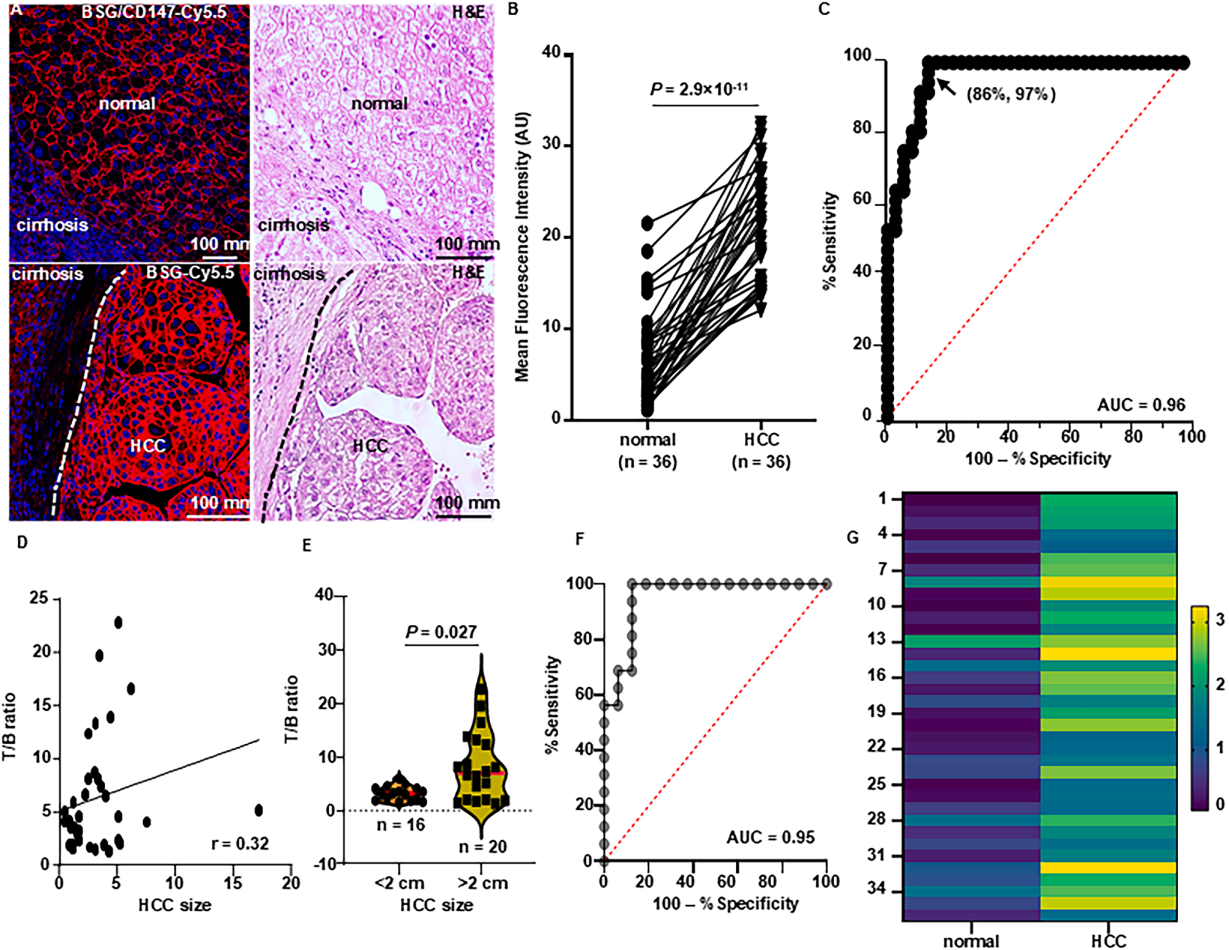
Ex vivo validation of BSG/CD147 expression in HCC. (A) Representative immunofluorescence (BSG/CD147-Cy5.5, red) and corresponding histology (H&E) of paired human liver tissues show markedly higher BSG/CD147 signal in HCC compared with adjacent cirrhotic and normal regions. (B) Quantified fluorescence intensities demonstrate significantly elevated T/B ratios in tumor relative to matched background (paired t-test, *P* = 2.9 × 10^−11^). (C) ROC analysis differentiated HCC from nontumor with high diagnostic performance (AUC = 0.96, 86% sensitivity, and 97% specificity). (D) Spearman correlation analysis reveals moderate positive association between tumor size and T/B ratio (ρ = 0.32, *P* = .04) to support increased BSG/CD147 expression with tumor progression. (E) T/B ratios in small (<2 cm) and large (>2 cm) HCC tumors shows significantly higher mean signal in larger lesions of 8.1 ± 1.4 vs 3.2 ± 0.32, *P* = .027. Violin plot shows greater signal variability among large tumors (CV = 77.7%) than smaller ones (CV = 40.2%). F) ROC curve analysis of small lesions demonstrates high diagnostic performance for distinguishing small HCC from background cirrhosis (AUC = 0.95; sensitivity 87%; specificity 93%). (G) Heatmap summarizes interpatient heterogeneity, and illustrates consistent elevation of BSG signal in HCC (<2 cm) vs background cirrhotic liver across n = 16 patients. BSG, Basigin; CD147, cluster of differentiation 147; CV, coefficient of variation; HCC, hepatocellular carcinoma; T/B, target-to-background.

BSG/CD147 expression in HCC was further validated by immunofluorescence staining of paired human liver specimens. Tumor regions exhibited markedly stronger signal intensity compared with adjacent cirrhosis or background liver, Figure 6A and B. ROC analysis demonstrated excellent diagnostic accuracy, area under the curve (AUC) = 0.96 with 86% sensitivity and 97% specificity, to distinguish HCC from background cirrhotic liver, Figure 6C. A weak positive correlation was observed between tumor size and protein expression, Figure 6D. Signal was further analyzed in HCC tumors <2 cm in diameter to assess its potential utility for early detection, Figure 6E. Despite the small size, these lesions showed clearly elevated fluorescence relative to background liver, Figure 6E. ROC analysis for nodules <2 cm demonstrated high diagnostic accuracy, AUC = 0.95 with 87.5% specificity and 93% sensitivity, Figure 6F. Although the mean T/B ratio was higher in larger tumors, the ability of BSG/CD147 to generate detectable contrast in <2 cm nodules underscores promise to identify early or borderline lesions that are otherwise difficult to classify radiologically (LI-RADS 3/4). Target expression also exhibited interpatient variability, Figure 6F, with the coefficient of variation of T/B ratios greater in large tumors (77.7%) than in small ones (40.2%) suggesting biological heterogeneity increases with tumor size, Figure 6E. Importantly, BSG/CD147 signal remained clearly detectable within small HCC foci embedded in cirrhotic tissue to support potential utility for distinguishing indeterminate liver nodules (<2 cm). In a representative specimen, strong fluorescence was observed in HCC tumor regions with only weak background signal in adjacent cirrhosis, Supplementary Figure 7A-D. A few isolated tumor cells within cirrhotic areas also demonstrated focal strong CD147 expression.

## Discussion

This study integrates single-cell transcriptomics, network analysis, and ex vivo validation to identify BSG/CD147 as a tumor-associated signaling hub and marker of hepatocyte state transitions in HCC. Using 2 independent scRNA-seq datasets, the transcriptional trajectory was reconstructed from normal hepatocytes through PT intermediates to malignant states, and a continuum of increasing stemness and oncogenic activation was identified. Multiple gene modules associated with tumor progression were found using hdWGCNA. The Hep-M20 module showed the strongest correlation with pathological stage. BSG/CD147 emerged from within this module as a key hub gene and exhibited progressive upregulation along pseudotime. Strong correlation with stemness potential implicates gene activation as an early molecular hallmark of hepatocarcinogenesis.

CD147 expression showed an overall increase in tumor tissues compared with nontumor liver. In bulk-level comparisons across clinical stages, the most pronounced elevation was observed in advanced stages (IIIB and IV), whereas differences among earlier stages (N, I, II, and IIIA) were less distinct. This suggests that CD147 expression is more strongly associated with advanced tumor burden at the tissue level. It should be noted that the number of normal samples was relatively small compared with tumor samples, which may limit the robustness of baseline comparisons. In our analysis, PT and tumor represent cell-type based classifications derived from single-cell annotation rather than clinical tumor staging. Therefore, differences between PT and T reflect cellular state transitions rather than early vs late clinical disease stages. Importantly, single-cell pseudotime analysis revealed a progressive increase in CD147 expression along the inferred tumor cell trajectory from normal hepatocytes to malignant states. This suggests that CD147 upregulation is associated with dynamic tumor cell state transitions during hepatocarcinogenesis. Taken together, while bulk-stage analysis indicates that CD147 expression is more pronounced in advanced-stage tumors, its gradual increase along the cellular trajectory and detectable expression in small tumors suggest that it may still be present at earlier disease stages, albeit with limited discriminatory power. Therefore, CD147 is better interpreted as a progression-associated biomarker rather than a strictly early-stage diagnostic marker.

CD147 (BSG) is encoded by multiple transcript variants, with BSG-2 representing the predominant and functionally relevant isoform in most tissues, including HCC.^24^ CD147 is known to mediate cyclophilin interactions and promotes tumor progression.^25^^,^^26^ At the protein level, CD147 also undergoes differential glycosylation, with the highly glycosylated form associated with enhanced protumorigenic activity.^27^ However, the scRNA-seq data used in this study do not provide sufficient resolution to distinguish between CD147 transcript isoforms; therefore, our analysis reflects overall BSG expression. In HCC, CD147 upregulation has been linked to hypoxia-driven signaling hypoxia-inducible factor 1 alpha (HIF-1α),^27^ oncogenic pathways; phosphoinositide 3-kinase/protein kinase B (PI3K/AKT) and mitogen-activated protein kinase/extracellular signal-regulated kinase (MAPK/ERK),^28^ and inflammatory signaling nuclear factor kappa B,^29^ as well as chronic HBV infection, which together may promote hepatocyte transformation. At the protein level, the highly glycosylated form (commonly referred to as BSG-2) is considered the dominant functional isoform driving tumor-promoting effects such as matrix metalloproteinase induction and extracellular matrix remodeling. To date, most studies in HCC have not resolved isoform-specific differential expression of CD147 at single-cell resolution, although BSG-2 is widely regarded as the principal functional isoform associated with tumor progression.

Consistent with prior work, our data suggest that CD147 may contribute to malignant transformation and progression through its involvement in tumor-associated signaling pathways. Expression correlated with CytoTRACE-derived stemness to suggest that CD147 upregulation accompanies hepatocyte dedifferentiation toward progenitor-like phenotypes known to promote tumor initiation and recurrence. Previous studies have shown that CD147 regulates metabolic reprogramming, extracellular matrix remodeling, and epithelial-mesenchymal transition through interactions with cyclophilins (PPIA/PPIB) and downstream MAPK/ERK, PI3K/AKT, and HIF-1α signaling cascades.^30^^,^^31^ Our single-cell and network analyses extended these observations by revealing that CD147-based signaling networks mediate cross-talk between tumor hepatocytes and fibroblasts, the 2 dominant cell types of the tumor microenvironment to form a cyclophilin-dependent tumor-stroma communication axis. Additionally, our findings suggest that BSG/CD147 can engage T cells through PPIA and PPIB ligands to establish an immunomodulatory communication channel that shapes the tumor-immune microenvironment. Our findings are consistent with the recent report that elevated CD147 expression in tumor cells is associated with an immunosuppressive tumor-immune microenvironment in HCC as evidenced by increased infiltration of regulatory T cells.^32^ This pathway likely fosters a microenvironment conducive to invasion, angiogenesis, and immune modulation, thereby integrating intrinsic tumor signaling with extrinsic stromal remodeling.^33^

Ex vivo validation further demonstrated that BSG/CD147 expression was significantly elevated in HCC compared with adjacent background liver, including in lesions <2 cm that are often radiologically indeterminate (LI-RADS 3/4). Our findings are consistent with the observations of a significantly elevated expression of CD147 in HCC compared to nontumor.^34^ ROC analyses demonstrated strong discriminatory performance in small tumors to support potential utility for detection of tumor-associated molecular changes in indeterminate lesions. Strong membrane localization, high T/B ratio, and minimal expression in surrounding cirrhosis or background liver establish a favorable foundation for development of targeted contrast agents. Incorporating BSG/CD147 assessment into HCC surveillance frameworks could substantially enhance performance for early diagnosis and enable biological risk stratification of indeterminate nodules. Unlike prior studies that largely evaluated CD147 expression in bulk tumor tissue or serum as a static diagnostic or prognostic marker,^35^, ^36^, ^37^ this work uses single-cell transcriptomics and pseudotime network modeling to demonstrate CD147 upregulation as an early event in hepatocyte transformation that is correlated with increasing stemness and malignant progression.

Despite the strengths of this integrated approach, several limitations should be noted. The sample size (n = 16) for small (<2 cm) lesions was modest, which may limit generalizability. In addition, the scRNA-seq datasets analyzed were annotated by pathological stage rather than derived directly from indeterminate nodules to restrict temporal resolution for early transformation events. Finally, while computational modeling identified plausible ligand-receptor and pathway interactions, further functional validation is needed to confirm the biological significance of BSG/CD147-mediated signaling in the tumor microenvironment.

## Conclusion

Taken together, these findings suggest that BSG/CD147 is well positioned as a marker of tumor-associated cellular state transitions and intercellular signaling, with potential utility for enhancing detection of tumor-associated molecular changes in early or indeterminate lesions in spatially resolved or targeted imaging contexts, rather than as a standalone bulk-level early-stage biomarker. Consistent expression in small, radiologically ambiguous lesions (<2 cm) highlights the translational potential of BSG/CD147 as a precision biomarker for early HCC detection and image-guided diagnosis. Integration of BSG/CD147-targeted strategies into molecular imaging or surveillance workflows could transform early HCC management by enabling detection, risk stratification, and intervention at the most curable stages of liver cancer.

## Figures and Tables

**Supplementary Figure 7:**